# Basic locomotor muscle synergies used in land walking are finely tuned during underwater walking

**DOI:** 10.1038/s41598-021-98022-8

**Published:** 2021-09-16

**Authors:** Hikaru Yokoyama, Tatsuya Kato, Naotsugu Kaneko, Hirofumi Kobayashi, Motonori Hoshino, Takanori Kokubun, Kimitaka Nakazawa

**Affiliations:** 1 Department of Electrical and Electronic Engineering, Tokyo University of Agriculture and Technology, Tokyo, Japan; 2 Japan Society for the Promotion of Science, Tokyo, 102-0083 Japan; 3 Department of Life Sciences, Graduate School of Arts and Sciences, The University of Tokyo, 3-8-1 Komaba, Meguro, Tokyo 153-8902 Japan; 4 College, National Rehabilitation Center for Persons with Disabilities, Saitama, 359-8555 Japan; 5 Department of Physical Therapy, Faculty of Health and Social Services, Saitama Prefectural University, Saitama, 343-8540 Japan

**Keywords:** Electromyography - EMG, Motor control, Central pattern generators

## Abstract

Underwater walking is one of the most common hydrotherapeutic exercises. Therefore, understanding muscular control during underwater walking is important for optimizing training regimens. The effects of the water environment on walking are mainly related to the hydrostatic and hydrodynamic theories of buoyancy and drag force. To date, muscular control during underwater walking has been investigated at the individual muscle level. However, it is recognized that the human nervous system modularly controls multiple muscles through muscle synergies, which are sets of muscles that work together. We found that the same set of muscle synergies was shared between the two walking tasks. However, some task-dependent modulation was found in the activation combination across muscles and temporal activation patterns of the muscle synergies. The results suggest that the human nervous system modulates activation of lower-limb muscles during water walking by finely tuning basic locomotor muscle synergies that are used during land walking to meet the biomechanical requirements for walking in the water environment.

## Introduction

A water environment serves as an alternative option to conventional therapy for active rehabilitation, which is termed hydrotherapy^1^. Hydrotherapy has been investigated as a form of therapy for individuals with various disorders, such as osteoarthritis^2^ and stroke^3^. Although numerous hydrotherapeutic exercises have been proposed, underwater walking is one of the most common hydrotherapeutic exercises^1,4^. With the growing popularity of underwater walking therapy, understanding the effects of the water environment on walking movements has been important for the construction of effective training regimens.

From a biomechanical point of view, the effects of the water environment on walking movement are mainly related to the hydrostatic and hydrodynamic theories of buoyancy and drag force. Greater depth of immersion increases the upthrust effect for body weight bearing due to buoyancy^1^. The force from buoyancy is also specific to movements in the vertical direction, with upward movements being assisted and downward movements resisting^5^. Drag force, on the contrary, is the resistance force that acts opposite to the movement direction and is related to the movement speed^6^. Furthermore, the effects of the fundamental physics principles of the water environment on walking movements have been investigated using kinematic, kinetic, and electromyographic (EMG) analyses^7–12^. Buoyancy decreases the vertical ground reaction forces to approximately one-third of the body weight when walking in chest-deep water^7,9^. When walking under water, there is no change in the ankle, knee, and hip range of motion, but extension torques considerably decrease in the ankle and knee joints compared to that walking on land^11^. Regarding muscle activity during underwater walking, previous studies on EMG activities reported lower activity of the calf muscles during the stance phase and larger activity of the rectus femoris (RF) and biceps femoris (BF) during the swing and stance phases, respectively, compared to those during land walking^9,10,13^.

**Figure 1:**
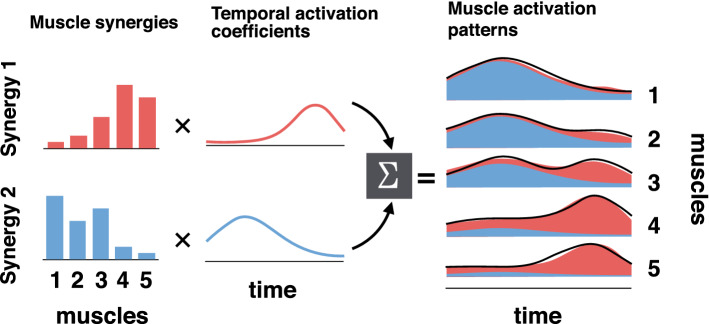
Schematic example of EMG reconstruction by the sum of activations of muscle synergies. The output of each muscle synergy (areas filled with blue or red in the right panel) is explained by the product of the muscle synergy (bars in the left panels; the activation level of each muscle) and the corresponding temporal activation coefficient (lines in the middle panels). Consequently, the total muscle activation (black lines in the right panel) is reconstructed by the sum of the muscle synergy activations (filled areas).

All previous studies on EMG during underwater walking have independently investigated the activity of each muscle^7–10,12,14^. However, it has been recognized that the human nervous system does not control all muscles individually but controls sets of muscles that work together, which are called muscle synergies, during walking^15–17^. Modular neuromuscular control through muscle synergies is widely accepted as an indicator of the coordination inherent in muscular control^18^. Muscle synergy represents a time-invariant activation combination across muscles, which are activated by a time-varying coefficient, and summation of activation of muscle synergies can reconstruct recorded muscle activation patterns (Fig. 1). Regarding the task specificity of locomotor muscle synergies, although whether the number of muscle synergies during locomotion changes depending on speed is still controversial, the temporal activation coefficients are modulated depending on speed^15,19–22^. Other studies have reported that similar muscle synergies are used for walking on different slopes, but the activation combination across muscles and the temporal activation coefficients are modulated depending on the conditions^23,24^. To date, no study has investigated the muscle synergies used during underwater walking. Thus, the details of modular control of the muscle activation patterns during water walking, which are different from those during land walking^7–10,12,14^, remains unclear. It was demonstrated that if the muscle synergies involved in different motor tasks are shared, the generalization of motor learning effects among tasks is maximized^25^. Therefore, investigation of the similarity of the muscle synergies between land and water walking may provide useful information for the neurorehabilitation of walking.

Therefore, we extracted muscle synergies from recorded EMG signals during land walking and water walking to examine modular neuromuscular control for walking in a water environment. Based on the two different adaptation patterns of muscle synergies to changes in walking conditions mentioned above^15,23^, we formulated the following two hypotheses for the adaptation of muscle synergies to the water environment: (1) muscle synergies specific to water walking are recruited, and (2) muscle synergies similar to those for land walking are used during water walking, but the activation combination across muscles and the temporal activation coefficients in the synergies are modulated.

## Materials and methods

### Participants

Eight healthy men (ages 21–32 years) with no history of neural or musculoskeletal disorders participated in the study. Each participant providedwritten informed consent for participation in the study which was performed in accordance with the Declaration of Helsinki and was approved by the local ethics committee of the Saitama Prefectural University Ethics Review Committee.

### Experimental setup and design

**Figure 2:**
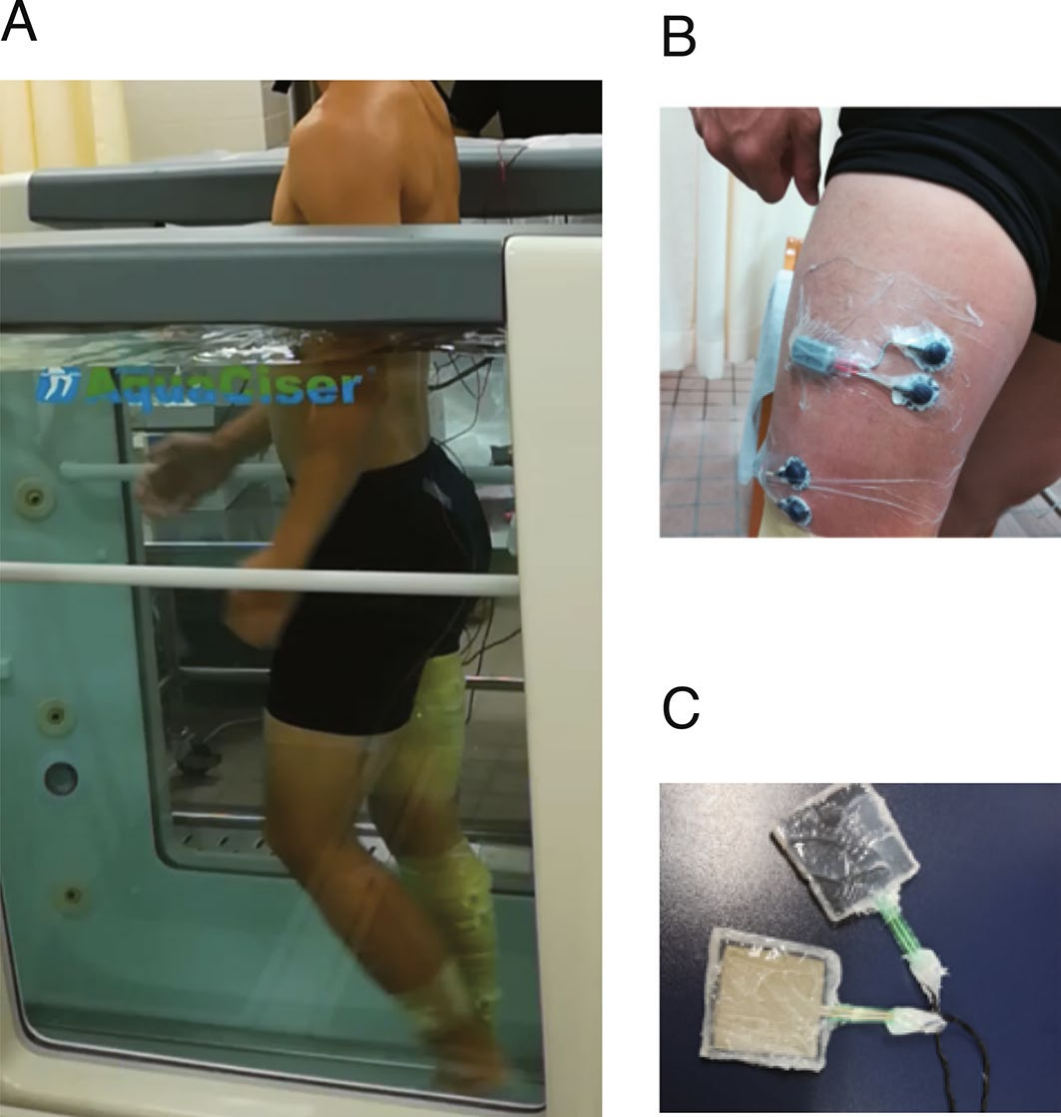
Experimental setup. (**A**) Lateral view of a participant walking in water condition. Participants walked on an underwater treadmill. (**B**) Waterproof electromyographic electrodes used in the present study. The waterproof electrodes were covered with waterproof adhesive sheets to prevent water immersion. (**C**) Force sensors (foot switches) were waterproofed with silicone coating.

The participants walked barefoot at 0.5 m/s for 2 min 30 s, first on an overground treadmill (Split belt treadmill, Bertec, USA), and then on an underwater treadmill (AquaCiser Underwater Treadmill System, Hudson Aquatic Systems LLC, USA) (Fig. 2A). Data collection was carried out for 2 min in the middle of the task to examine steady-state walking by removing the first and last 15 s of the task. The same walking speed was used for both land and water conditions to evaluate the effects of the water environment on walking by removing speed-dependent effects following previous studies^12,26^. The walking speed was chosen based on previous studies on underwater walking^9,11,12^. The water depth was set corresponding to the xiphoid process level for each participant. The water temperature was maintained at 34 °C throughout the experiment.

### Data collection

Surface electromyographic (EMG) signals were recorded from the following 10 leg muscles on the right side using a wireless EMG system (Mini Wave Infinity Waterproof, Cometa Systems Inc., Italy): gluteus maximus (Gmax), gluteus medius (Gmed), tensor fasciae latae (TFL), vastus lateralis (VL), RF, BF, semitendinosus (ST), tibialis anterior (TA), soleus (SOL), and medial gastrocnemius (MG). EMG signals were amplified (with a 1000 gain preamplifier), band-pass filtered (10–500 Hz), and sampled at 1000 Hz. We covered the waterproof EMG electrodes with waterproof adhesive sheets to prevent water immersion (Fig. 2B). Silicone-coated waterproof foot-switches (size:44.0 mm × 44.0 mm × 1.2 mm) were attached to the heel to determine the time of foot contact (Fig. 2C).

### Data analysis

The recorded EMG data were high-pass filtered with a zero-lag Butterworth filter (fourth-order, a cut-off frequency of 30 Hz), demeaned, full-wave rectified, and smoothed with a zero-lag Butterworth low-pass filter (fourth-order, a cut-off frequency of 4 Hz) to obtain EMG envelopes^27,28^. We resampled the EMG envelopes at 100 Hz. The amplitude of the EMG envelopes for each muscle was normalized to the maximum value for that muscle through the two-condition walking tasks. Then, we extracted muscle synergies from the EMG envelopes by non-negative matrix factorization (NNMF)^15,16^. Muscle synergies were extracted in each task for each participant from the EMG dataset organized as a matrix with 10 muscles × 12,000 variables (i.e., 100 Hz × 120 s [2 min]). The EMG matrix (*M*) was decomposed into spatial muscle weightings (*S*), which is referred to as the muscle synergies and their temporal activation patterns (*C*) by NNMF according toequation (1):

1 $$ M = S \cdot C + E_{ } $$

where *M* (*m* × *t* matrix, where *m* is the number of muscles^10^, and *t* is the number of samples [12,000]) is a linear combination of muscle synergies, *S* (*m* × *N*_*synergy*_ matrix, where *N*_*synergy*_ is the number of muscle synergies), and their temporal activation patterns, *C* (*N*_*synergy*_ × *t* matrix), and *E* is the residual error matrix. For comparisons among participants and walking tasks, the weightings of each muscle synergy and temporal activation patterns were normalized so that the individual muscle weighting vector was a unit vector as previously suggested^29,30^.

The optimal number of muscle synergies, *N*_*synergy*_, was determined by iterating each possible synergy from 1 to 7. We calculated the variance accounted for (VAF)^31^ to examine the goodness of fit between the original EMG and reconstructed EMG from muscle synergies at each *N*_*synergy*_. Considering the local minima inherent in NNMF, the muscle synergy extraction was iterated 10 times for each possible *N*_*synergy*_ from 1 to 7, and VAF was calculated at each iteration. The iteration with the highest VAF was maintained. We then selected the least number of muscle synergies that accounted for > 90% of VAF^28,31^ while adding an additional synergy did not increase VAF by > 5%^28,32^. To facilitate the comparison of the set of muscle synergies among participants, we used the same number of muscle synergies as the rounded mean number of synergies across subjects, that is, 4 in both tasks (mean numbers: 4.00 ± 0.76 (mean ± standard deviations **[**SD]) and 4.13 ± 0.99 for land and water conditions, respectively), for further analyses^30^. Then, we sorted the extracted muscle synergies into four groups in each condition based on muscle weightings using the k-means method implemented by MATLAB (“kmeans” function, MathWorks, Inc., Natick, MA, USA)^33^.

Some previous studies have evaluated how well a set of muscle synergies in one condition reconstructed muscle activities in other conditions to evaluate the similarity of the synergies among different conditions^15,27,34–36^. Using this test, we tested the similarity of the synergies between the two walking conditions. In this test, we reconstructed EMG activity during water walking using the NNMF algorithm initialized with the muscle synergies extracted during the land walking condition and updating only to the temporal patterns of the synergies to reduce the error between the original and reconstructed EMG^15,27,34–36^. We then calculated the VAF to examine the goodness of fit between the original and reconstructed EMG.

### Statistics

We compared the temporal activation patterns of muscle synergies for each type between the two walking conditions. First, we divided the activation patterns in a gait cycle into 20-time points (i.e., 1–5%, 6–10%, …, 96–100%) and calculated the averaged values in each bin. Then, the values at each time point between the two conditions were compared using a paired permutation with 1000 random permutations^37^, which is a non-parametric paired comparison method because the normal distribution was not observed in all cases (tested using the Lilliefors test). The p values obtained from the permutation test were corrected using the false discovery rate (FDR) correction for multiple comparisons^38^. We also compared the temporal activation patterns of each muscle using the same procedures as the comparisons for temporal activation patterns of muscle synergies.

Regarding activation combination across muscles (i.e., construction of muscle synergies), we compared weightings for each muscle in each synergy type between the two conditions using the paired permutation test with FDR correction. In the muscle weightings, a normal distribution was not observed in all cases (tested by the Lilliefors test). We also examined the similarity of muscle weightings and temporal patterns in each type between the conditions based on Spearman correlation coefficients, which is a non-parametric correlation measure. We calculated the correlation coefficients of muscle weightings and temporal patterns for each synergy type for each participant. The number of muscle synergies between the two conditions was also compared by the paired permutation test because the data were not normally distributed (tested by the Lilliefors test).

## Results

### EMG signals during land walking and water walking

**Figure 3:**
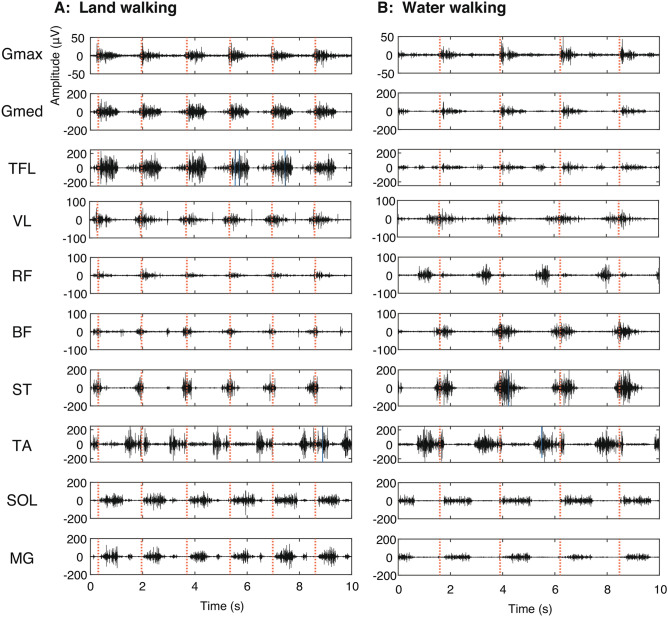
Examples of electromyographic (EMG) signals during land walking (**A**) and water walking (**B**) from a participant. EMG signals were recorded from ten muscles of the right leg. Vertical red dashed lines indicate right foot-contact timings.

Figure 3 shows typical examples of recorded EMG signals of 10 leg muscles from a subject during land and water walking. Based on visual inspections, there were no clear artifacts in the recorded EMG signals both during land and water walking. Evident water environment-dependent changes in the EMG patterns based on visual inspection in the examples were as follows: 1) decreased amplitude in the Gmed and TFL, 2) an activation timing shift from after the foot contact to before the foot contact in the RF, and 3) decreased amplitude modulation in the MG.

**Figure 4:**
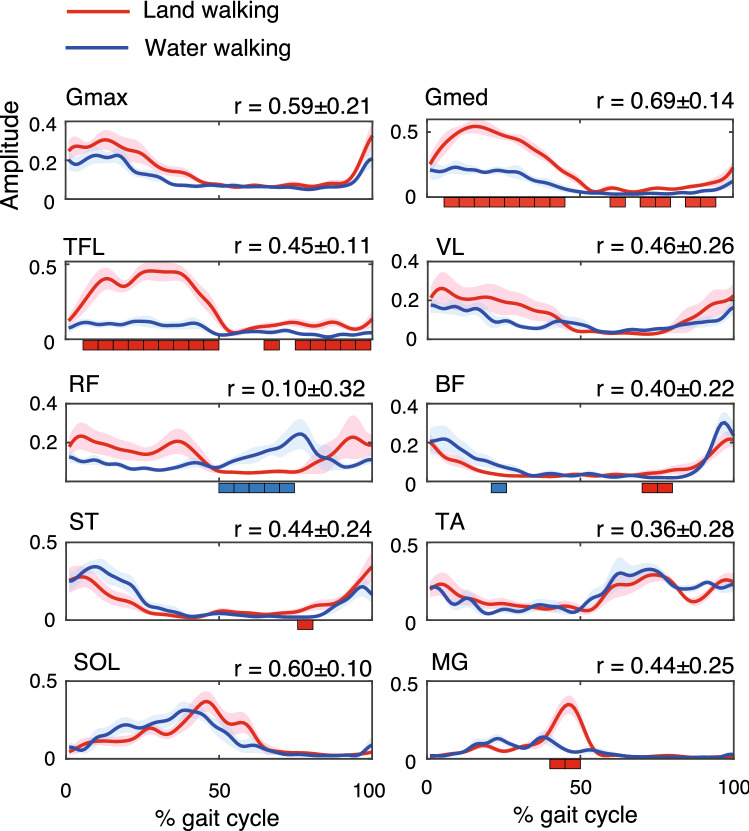
Group means with standard errors (SE) of muscle activation patterns during land walking (red) and water walking (blue). Red and blue rectangles under the plots indicate timing at which a significant difference existed between the two tasks (*p* < 0.05). Red rectangles mean that the activation was greater in land-walking compared to that in water-walking. Blue rectangles mean that it was greater in water-walking than land-walking. Group means with standard deviations (SD) of the correlation coefficients between the two tasks are shown above the plots.

Figure 4 shows the group-averaged envelopes of the EMG signals. The group-level results statistically supported the above-mentioned water-environment-dependent changes in the muscle activation patterns. Group means of correlation coefficients of the EMG envelopes between the conditions showed moderate correlations in most of the muscles (r = 0.40–0.69), while RF and TA showed low correlation values (r = 0.10 and 0.36, respectively).

### Muscle synergies during land walking and water walking

**Figure 5:**
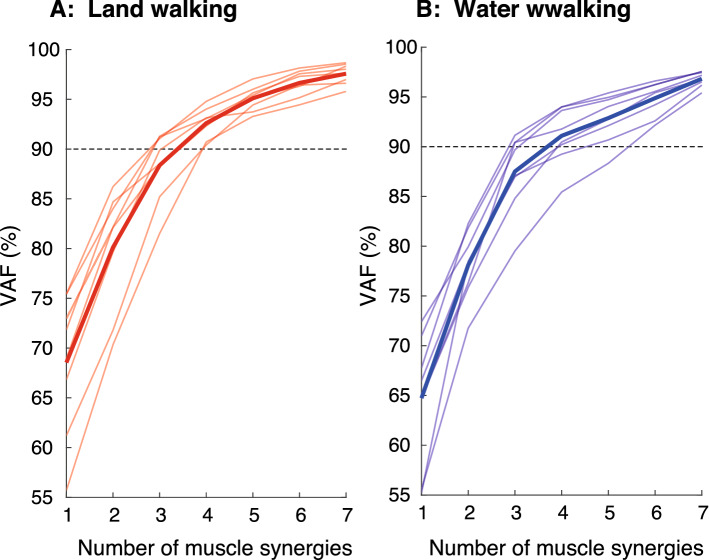
Individual (thin lines) and participant mean (thick lines) of the percentage of Variability Accounted For (VAF). Left and right panels indicate VAF for land-walking and water walking, respectively. Horizontal dashed lines indicate the threshold to determine the number of extracted muscle synergies.

Figure 5 shows the VAF at each number of muscle synergies in each condition. Extracted number of synergies were 4.0 ± 0.76 (mean ± SD) and 4.13 ± 0.99 for land walking and water waking conditions, respectively. There was no significant difference in the number between the conditions (*p* > 0.05).

**Figure 6:**
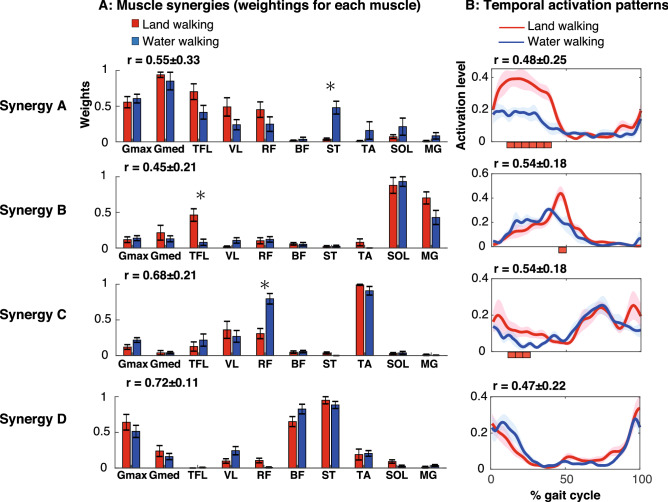
Construction of muscle synergies (weightings for muscles) (**A**) and their temporal activation patterns (**B**). (**A**) Bar plots indicate participant mean and standard error (SE) of the weightings of each muscle. Asterisks associated with certain muscles indicate significant differences in the weightings between the two tasks (*p* < 0.05). Correlation coefficients between the two tasks are shown above the bar plots. (**B**) Line plots indicate participant mean and SE of the temporal patterns of muscle synergies. Red rectangles under the line plots indicate timing when activation during land-walking was significantly larger than that during water-walking (*p* < 0.05). Correlation coefficients between the two tasks are shown above the plots.

Figure 6 shows four different types of muscle synergies (Fig. 6A) and the corresponding activation coefficients (Fig. 6B). The four types of muscle synergies were generally similar between the conditions (group means of correlation coefficients: 0.45 < r < 0.72), but some statistical differences were found in Synergies A–C.

Synergy A mainly represented the activation of the Gmax, Gmed, TFL, VL, and RF in both conditions and the ST only in the water walking condition (*p* < 0.05). The synergy worked in the early to mid-stance phase (approximately 1 − 40% of the gait cycle) in both conditions, but the activation was smaller in 11–40% of the gait cycle during water walking than during land walking (*p* < 0.05).

Synergy B mainly represented the activation of the SOL and MG in both conditions and TFL only during land walking (*p* < 0.05). The synergy mainly worked in the mid to late stance phase (16–55% of the gait cycle) in both conditions, but the activation was smaller in 46–50% of the gait cycle during water walking than during land walking (*p* < 0.05).

Synergy C showed the higher activation of TA, VL, and RF in both conditions. However, the weighting for RF was significantly larger for water walking than for land walking (*p* < 0.05). The synergy mainly worked in two timings: (1) the mid-swing phase (approximately 66–80% of the gait cycle) and (2) the late swing phase to the mid-stance phase (approximately 90–35% of the gait cycle). The activation at the latter timing was smaller in 11–25% of the gait cycle during water walking than during land walking (*p* < 0.05).

Synergy D showed higher activation of Gmax, BF, and ST under both conditions. This synergy mainly worked during the late swing and early stance phases (approximately 95 − 20% of the gait cycle). No significant differences were found in the muscle weightings and temporal patterns.

We also tested the similarity of the synergies between the two walking conditions by decomposing the EMG patterns of underwater walking by imposing the same set of synergies found during land walking. The VAF of the reconstructed EMGs during water walking by the muscle synergies during land walking was 84.3 ± 5.6% (mean ± SD).

## Discussion

We found that four muscle synergies were recruited during both land and water walking (Fig. 6). Although the extracted muscle synergies were similar between the tasks, some task-dependent differences were found in the construction of muscle synergies (i.e., muscle weightings) and temporal activation patterns (Fig. 6). The results support our second hypothesis and suggest that lower-limb muscles were modularly controlled with a similar set of muscle synergies during land and water walking, but the activation patterns of the muscle synergies were modulated to fit the water environment.

### Robustness of the extracted muscle synergies between land and water walking

Although some minor task-dependent differences were found, the extracted muscle synergies were generally similar between land and water walking (Fig. 6). Another result, that muscle synergies extracted during walking on land can explain more than 80% of the variance in EMG during walking in water (VAF = 84.3 ± 5.6%), also supports the similarity of muscle synergies. Contrary to the robustness of locomotor muscle synergies across conditions in this study, some previous studies have reported task specificity in muscle synergies regarding locomotion speed^15,19,39^. Studies have found that a lower number of muscle synergies are extracted during very slow walking (< 0.6 m/s) compared to during comfortable speed walking^15,19^ and different types of muscle synergies are used between walking and running^15,39^. Speed- and mode-dependent modular control of locomotor muscle activity was revealed in spinal neuronal networks in mice^40^ and may be phylogenetically conserved in the human nervous system. However, other studies showed the same number of muscle synergies were extracted regardless of speed^20–22^. The discrepancy among the previous studies may be related to differences in the analyzed muscle sets and the range of speeds. Thus, it should be kept in mind that whether the number of muscle synergies during locomotion changes depending on speed is still controversial.

At the same speed, a similar set of lower limb muscle synergies was extracted during slope walking^23,24^, walking with Nordic poles^30^, and walking on an uneven surface^41^ as that during normal walking. Similar to these previous studies, our results also showed the similarity of the extracted muscle synergies between land and water walking at the same speed. Thus, when walking in different environments, the human nervous system may use basic locomotor muscle synergies and modulate the activation patterns to fit the situation, except for drastic changes such as 95% body weight support^17^, or sudden translation perturbations of the walking surface^42^.

### Fine tuning of muscle synergies to adapt to the water environment

The extracted muscle synergies were similar between land and water walking, but some task-dependent differences were found in the construction of muscle synergies (i.e., the muscle weightings) and the temporal activation coefficients (Synergies A−C in Fig. 6).

Synergy A mainly represented the activation in the early to mid-stance phase of the Gmax, Gmed, TFL, VL, and RF in both conditions and the ST only during water walking (Fig. 6). The ST works for knee flexion with internal rotation and hip extension. Thus, the added ST activity in Synergy A may contribute to larger hip extension and knee flexion torques in the stance phase during water walking than land walking^11^. This synergy also showed another task-dependent difference in that the temporal activation was smaller in 11–40% of the gait cycle during water walking than during land walking (Fig. 6). Because the muscles, which are largely involved in the synergy, work for the loading response in the stance phase, the bodyweight reduction effect by buoyancy in the water environment certainly decreased the temporal activation in the stance phase during water walking.

Synergy B mainly represented the activation in the mid to late stance phase of the SOL, MG, and TFL during land walking (Fig. 6). However, the weighting for the TFL was smaller for water walking than for land walking (Fig. 6). TFL activation during the late stance phase was previously reported during land walking^43^. This TFL activity during land walking contributes to lateral rotation of the pelvis during the late stance prior to the double support^44^. A previous study reported that the lateral rotation movement of the pelvis during the late stance is smaller in water walking (from approximately − 5° to 0°) compared to that during land walking (from approximately − 10° to 10°)^45^. The small lateral rotation during water walking may be related to the small weighting of the TFL in Synergy B during water walking. In addition to the weighting of TFL, this synergy also showed a task-dependent difference in that the temporal activation was smaller in 46–50% of the gait cycle during water walking than during land walking (Fig. 6). The muscles primarily activated in Synergy B were the plantar flexors (SOL and MG). The activation decrease of Synergy B during the late stance phase would be related to lower ankle extension torque at late stance during water walking than land walking^11^. The water environment reduces the friction between the foot and the treadmill surface owing to buoyancy because friction is proportional to the force perpendicular to the contact surface. It is possible that the plantar flexors could not generate a forward propulsive force as they do on the ground because of the lower frictional force from the contact surface. This may have caused the small activation of Synergy B in the late stance during water walking.

In land walking, Synergy C represented higher activation of the TA with moderate activation of the VL and RF at two timings: (1) the mid-swing phase and (2) the late swing phase to the mid-stance phase (Fig. 6). The weighting for the RF was larger for water walking than for land walking, and the temporal activation at the latter timing was smaller in 11–25% of the gait cycle during water walking than during land walking (Fig. 6). It is probable that the increased weighting for the RF is related to the increased drag force against the forward movement of the leg during the swing phase in water. Regarding the possible role of the RF in the swing, a previous study reported a larger hip flexion torque during the mid-swing phase during water walking than during land walking^11^. The smaller activation in the 11–25% gait cycle during water walking than during land walking (Fig. 6), probably due to the bodyweight reduction due to buoyancy, which mainly affects the VL and RF activities, which are involved in body weight acceptance.

In the present study, only Synergy D did not show any significant differences in muscle weightings and temporal patterns (Fig. 6). This synergy represented the higher activation of Gmax, BF, and ST in the late swing and early stance phases (approximately 95–20% gait cycle). Synergy activation works for the deceleration of the leg in the late swing and acceleration of the leg in the early stance^46^.

### Methodological consideration

In this study, we used treadmills for both land and water walking. Since the participants walked almost in place during treadmill walking, the effects of drag force in the water environment during treadmill walking were less than those during walking across the pool. In this study, we found an additional involvement of the RF in Synergy B, which works for leg acceleration in the swing, due to the drag force in the water environment during treadmill walking (Fig. 6). However, given the difference in drag force, the involvement of the RF would be even larger during walking across the pool than during treadmill walking in water.

It should be considered how the preferred walking speed is affected by a water environment because the optimal walking speed is highly affected by gravitational levels, which corresponds to the amount of unloading^47^. To examine preferred walking patterns under different gravitational conditions, an approach of varying the treadmill speed depending on the amount of unloading based on the Froude number, referred to as dimensionless speeds, has been used^48,49^. The Froude number (Fr) is derived from a traditional inverted pendulum walking model and is defined as follows:

2 $$ Fr = V^{2} /g \cdot L $$

where V is the gait speed, g is the gravitational acceleration, and L is the leg length^48,50,51^. If two gait motions have equal Froude numbers, the two gait patterns are dynamically similar even in the two walking motions at different gravitational levels or leg lengths^50^. The present study used the same speed (0.5 m/s) in the land and water walking conditions. However, based on the Froude number, the walking speed corresponding to 0.5 m/s underwater would be approximately 0.9 m/s during land walking, considering that buoyancy decreases the vertical ground reaction forces to approximately one-third of the bodyweight when walking in chest-deep water^7,9^. Therefore, the dynamic conditions for walking were different for the two walking conditions. Thus, the possibility that the reported differences in muscle synergies may be due to the different Froude numbers between the two walking conditions. However, increased activity of RF and decreased activity of Gmed, Gmax, and TFL were observed as representative changes of muscle activity in water walking at 0.5 m/s compared to land walking at 0.5 m/s (Fig. 4). Such muscle activity changes compared to land walking at 0.5 m/s were not found in land walking at 0.9 m/s^15^, which has approximately the same Froude number as water walking at 0.5 m/s. Therefore, differences in optimal walking speed based on the Froude number probably did not explain all the differences in muscle synergy patterns found in this study. Future studies should compare muscle synergies during land and water walking with the same Froude number to examine the differences in muscle synergies derived from different Froude numbers or water environment-dependent modulation.

We measured EMG signals from the muscles on the right leg. However, we did not control leg dominance of the participants. We should have controlled the leg dominance, given the difference in characteristics between the dominant and non-dominant sides.

## Conclusions and perspectives

We found that a similar set of muscle synergies was used between land and water walking. However, the activation combination across muscles and the temporal activation coefficients in the muscle synergies were finely modulated mainly because of the effects of buoyancy and drag force by the water environment. Thus, the results of this study suggest that the human nervous system modulates activation of lower limb muscles with a basic set of locomotor muscle synergies during water walking and finely tunes their activation to meet the biomechanical requirements for walking under water.

Because underwater walking does not alter the coordination patterns of the lower limb muscles, it is suggested that underwater walking can be a suitable rehabilitation exercise. It reduces the gravitational load on joints when compared to land walking for the elderly and patients with impaired posture or locomotion. In upper-limb movement, it was demonstrated that if the muscle synergies involved in different motor tasks are shared, the generalization of motor learning effects among tasks is maximized^25^. Conversely, as different synergies are recruited, generalization is reduced or lost^25^. Based on the previous study^25^, the high similarity of the muscle synergies between land and water walking suggests that the motor learning effects during underwater walking may be highly generalized to land walking. However, it should be noted that the findings of this study was obtained from healthy participants. Thus, generalization of the results to the patients and the elderly needs to be considered carefully because their motor strategies are different from those of healthy individuals.

## Data Availability

The datasets analyzed during the current study are available from the first or corresponding author on reasonable request.
